# Autoimmune Bullous Disease Quality of Life (ABQoL) questionnaire: Validation of the translated Persian version in pemphigus vulgaris

**DOI:** 10.1016/j.ijwd.2020.03.043

**Published:** 2020-03-26

**Authors:** Amir Teimourpour, Kowsar Hedayat, Fereshteh Salarvand, Narges Ghandi, Maryam Ghiasi, Hamidreza Mahmoudi, Kamran Balighi, Roja Toosi, Farnam Mohebi, Ali Nili, Maryam Daneshpazhooh, Dedee F. Murrell, Cheyda Chams-Davatchi

**Affiliations:** a Autoimmune Bullous Diseases Research Center, Tehran University of Medical Sciences, Tehran, Iran; b Department of Epidemiology and Biostatistics, School of Public Health, Tehran University of Medical Sciences, Tehran, Iran; c Non-Communicable Diseases Research Center, Endocrinology and Metabolism Population Sciences Institute, Tehran University of Medical Sciences, Tehran, Iran; d Department of Dermatology at St. George Hospital, University of New South Wales, Sydney, Australia

**Keywords:** Validity, Reliability, Autoimmune Bullous Disease Quality of Life, Autoimmune bullous diseases, Pemphigus vulgaris, Farsi, Persian

## Abstract

**Background:**

Autoimmune bullous diseases are a group of rare, chronic, blistering diseases, with pemphigus vulgaris (PV) being the most common type in Iran. Skin and mucosal involvement and therapy may have a dismal impact on the quality of life of affected patients.

**Objective:**

We aimed to assess the validity and reliability of the Farsi (Persian) version of the Autoimmune Bullous Quality of Life (ABQoL) index in Iranian patients with PV.

**Methods:**

Forward and backward translation procedures were used to prepare the Persian version of the ABQoL questionnaire. A total of 180 patients were asked to complete the questionnaires, and 24 cases repeated the test after 2 weeks. For validity and reliability, an exploratory factor analysis was performed along with a parallel analysis to determine the number of factors. The multi-trait, multi-method matrix assessed convergent and discriminant validity. To evaluate internal reliability and reliability over time, Cronbach's alpha and intraclass correlation coefficients were reported.

**Results:**

Two factors explained a total variance of 54.9% in the data. The first and second factors comprised questions 1–3, 5, 7, 9, and 12–17 (symptoms-social) and questions 4, 6, 8, 10, and 11 (mucosal-psychological), respectively. The internal reliability of the Persian version of the ABQoL index was also acceptable, with a Cronbach's alpha of .855 for total items, .918 for the first factor, and .6 for the second factor. Our results suggest an acceptable convergent and discriminant validity of the Persian version of the ABQoL questionnaire.

**Conclusion:**

The Persian version of ABQoL index is a valid and reliable tool to monitor quality of life in patients with PV. Further studies are needed to assess the sensitivity of this instrument to changes in different disease activity and correlation with more general tools for the measurement of quality of life.

## Introduction

Autoimmune bullous diseases (AIBDs) are a group of rare, chronic, blistering diseases that are characterized by the presence of IgG autoantibodies against cell–cell adhesion of keratinocytes. Pemphigus vulgaris (PV) constitutes the majority of cases of AIBDs in some countries, with an incidence that varies in different parts of the world. In Iran, which is recognized as a high-incidence area, PV is estimated to affect 1 to 1.6 in every 100,000 people (Chams-Davatchi et al., 2005, Daneshpazhooh et al., 2012). PV has variable clinical presentations, severity, and epidemiologic characteristics, but usually appears as flaccid blisters on the mucosae and skin that rupture easily, leading to painful erosions (Chams-Davatchi et al., 2005). The unpleasant appearance (e.g., facial lesions) and symptoms (e.g., pain, itching, or burning) of skin involvement and problems during eating and drinking caused by oral mucosal erosions leave a significant impact on patients' quality of life (QoL). Furthermore, the long-term systemic steroids and immunosuppressants prescribed to control the disease cause side effects that may further worsen patients' QoL.

To date, a large body of evidence exists about the QoL of patients with blistering diseases. Nevertheless, much of the literature regarding the deterioration of the QoL by AIBDs have used general health questionnaires (Arbabi et al., 2011, Tabolli et al., 2008) or general dermatologic instruments, such as the Dermatology Life Quality Index (DLQI; Ghodsi et al., 2012). Although these tools are useful for the comparison of the impact of AIBDs with other chronic diseases or other dermatologic disorders, they have shortcomings. An appropriate tool for QoL in dermatology should show changes in different stages of the disease and the efficacy of various treatment strategies. By developing a disease-specific instrument, each domain of the QoL can be assessed more deeply, an issue that is not possible with the more general tools mentioned. With an in-depth assessment, any change in QoL would be more important in guiding treatment. Thus, disease-specific instruments are theoretically more sensitive and specific (Finlay et al., 1990).

In 2012, Murrell et al. introduced the first disease-specific instrument for QoL in patients with blistering diseases, called the Autoimmune Bullous Disease Quality of Life (ABQoL) questionnaire (Sebaratnam et al., 2013). The original version is a 17-item, self-reported, English language questionnaire with a moderate correlation to DLQI and Short Form 36 in convergent validity and is more sensitive than the DLQI in discriminant validity. Since its development in an Australian patient cohort, the reliability and internal consistency of the ABQoL index have also been confirmed in North American, Greek, Polish, Chinese, and Egyptian/Tunisian populations to date (Kalinska-Bienias et al., 2017, Patsatsi et al., 2017, Saleh et al., 2019, Sebaratnam et al., 2015, Yang et al., 2017). The ABQoL has recently been shown to be a useful tool in clinical trials for patients with PV and mucosal involvement (Krain et al., 2019). Thus, we aimed to assess the validity and reliability of the Farsi (Persian) version of the instrument in Iranian patients with PV.

## Methods

The Persian version of the ABQoL questionnaire was prepared by forward and backward translation into Farsi and back to English, procedures established by the World Health Organization guidelines (World Health Organization, 2010).

A total of 180 patients with a confirmed diagnosis of PV and clinically active disease who were seen at the Autoimmune Bullous Diseases Clinics of Razi Hospital in Tehran, Iran were recruited in the study. Informed consent was obtained from all participants, and the study was approved by the ethics committee.

The diagnosis was based on clinical (skin and/or mucosal bullae/erosions), histopathological (suprabasal clefts and acantholysis), immunofluorescence (intercellular IgG and/or C3 deposits), and serological findings (presence of anti-desmoglein 3 ± 1 by enzyme-linked immunosorbent assay). Patients age < 18 years and those who were unable to read and understand the questionnaire were excluded. Patients were asked to complete the questionnaire. Twenty-four repeated the test after 2 weeks.

For validity and reliability purposes, an exploratory factor analysis (Kaiser-Meyer-Olkin [KMO] index and Bartlett's test) was performed along with a parallel analysis to determine the number of factors. The acceptable value is > .5. Convergent and discriminant validity were assessed with a multi-trait, multi-method matrix (Field, 2013). Little's Missing Completely At Random test was used to evaluate the assumption that missing values are completely random (Little, 1988). Parallel analysis and multi-trait, multi-method matrix were performed by using the psy package in R software (Falissard, 2011), and other statistical analyses were conducted in SPSS, version 24.0 (IBM Corp.; Armonk, NY). To evaluate internal reliability and reliability over time, Cronbach's alpha and intraclass correlation coefficients were reported, respectively (Koo and Li, 2016). Independent t-test and Pearson correlation coefficient were used whenever applicable.

## Results

The questionnaire was filled out by 180 patients (123 women [68.3%]). The median age was 45 years (range, 20–85 years). All patients had active disease. The mean total score was 29.37 in our patient population.

With regard to the missing values, Little's Missing Completely At Random test was performed, and the results were not significant (χ^2^ = 319.640; degrees of freedom = 317; *p* = .447). Thus, the null hypothesis that the missing values were completely at random could be accepted.

**Fig. 1:**
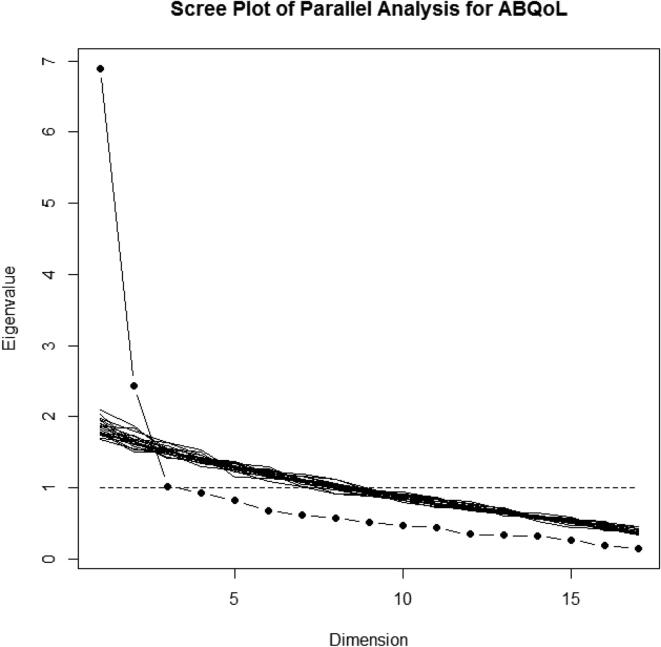
Parallel analysis of scree plot for Autoimmune Bullous Disease Quality of Life questionnaire with 17 items with 20 simulations suggested two factors.

Parallel analysis of the scree plot revealed two factors for the ABQoL questionnaire with 17 items and 20 simulations (Fig. 1). By assuming two factors, the results of the factor analysis with the principal component analysis extraction and Promax with Kaiser normalization methods are reported in Table 1. These two factors explained the total variance of 54.9% in the data. The KMO measure of sampling adequacy was .855, and Bartlett's test of sphericity was significant (χ^2^ = 780.05; df = 136; *p* < .001), indicating the usefulness of performing the factor analysis. None of the KMO values for each item was <.661.

**Table 1:** Factor loading of explanatory factor analysis by considering principal component analysis as extraction method and Promax with Kaiser normalization as rotation method.

	Factor	
	1	2
Question 1	**.619**	.110
Question 2	**.745**	.025
Question 3	**.709**	−.023
Question 4	−.388	**.416**
Question 5	**.805**	−.130
Question 6	−.056	**.756**
Question 7	**.647**	.018
Question 8	−.134	**.814**
Question 9	**.650**	.296
Question 10	.064	**.733**
Question 11	.212	**.748**
Question 12	**.486**	.249
Question 13	**.695**	−.045
Question 14	**.887**	−.131
Question 15	**.777**	.157
Question 16	**.771**	−.073
Question 17	**.776**	−.164

Items in bold identify the factor to which the item was assigned.

**Fig. 2:**
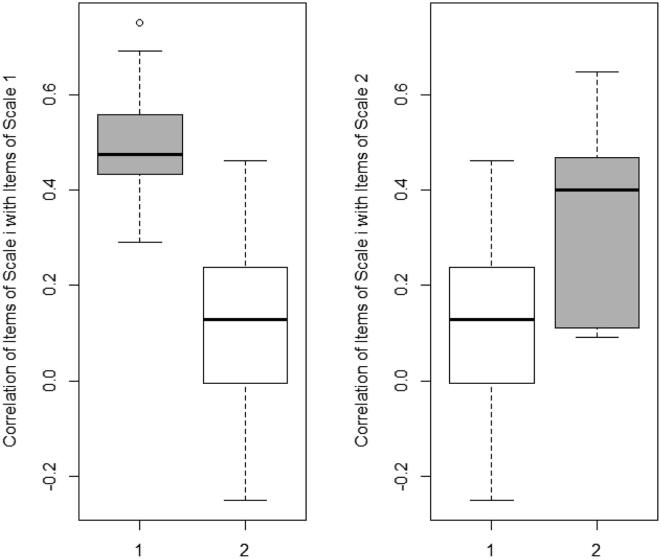
Distribution of inter-item correlations for the Persian version of Autoimmune Bullous Disease Quality of Life factors. The corrected correlation of items with their scale or factor (the gray boxes) is above the correlation of items with the other scale or factor (the white boxes). 1 F1 and 2 F2 stand for factor one and factor two.

Based on the factor loading of Table 1, the first and second factors comprised questions 1–3, 5, 7, 9, and 12–17 (symptoms-social) and questions 4, 6, 8, 10, and 11 (mucosal-psychological), respectively. For convergent and discriminant validity, the results of the multi-trait, multi-method approach are shown in Table 2 and Fig. 2. In Table 2, the correlation of each item with the corrected total score of each factor is reported. The corrected correlation of each item with its factor was clearly higher than the correlation with another factor, except for question 4 (slow skin healing). Although this item loaded on the second factor, it was not convergent to other items within this factor. The results are summarized as a boxplot in Fig. 2 for a global overview. The different items correlated better with items from the same factor. Our results suggest an acceptable convergent and discriminant validity of the Persian version of the ABQoL questionnaire.

**Table 2:** Corrected interitem correlations for Persian version of Autoimmune Bullous Quality of Life questionnaire factors.

	Item	Item scale	Factor 1	Factor 2
1	Question 1	1	.595	.207
2	Question 2	1	.708	.166
3	Question 3	1	.66	.118
4	Question 5	1	.668	.015
5	Question 7	1	.602	.137
6	Question 9	1	.675	.346
7	Question 12	1	.512	.279
8	Question 13	1	.647	.118
9	Question 14	1	.82	.04
10	Question 15	1	.769	.262
11	Question16	1	.705	.077
12	Question 17	1	.69	.027
13	Question 4	2	−.201	.183
14	Question 6	2	.154	.52
15	Question 8	2	.097	.614
16	Question 10	2	.295	.531
17	Question 11	2	.418	.602

The internal reliability of the Persian version of the ABQoL questionnaire was also acceptable, with Cronbach's alpha of .855 for total items, .918 for the first factor, and .6 for the second factor. After deleting the fourth quarter, the Cronbach's alpha of the second factor increased to .721. The intraclass correlation coefficient value for test–retest reliability was .885. Women had a higher total score in comparison to men (31.05 ± 9.19 vs 25.75 ± 12.1, *p* < .001): Symptoms-social score was higher (23.1 ± 10.5 vs 17.46 ± 9.7, *p* < .001) but not mucosal-psychological (*p* > .5). There was no correlation between age and total score and the two factors (all *p* > .2).

## Discussion

In this study, we carried out validity and reliability tests for the ABQoL questionnaire in an Iranian patient cohort with a blistering autoimmune disease, namely PV. The mean total score (29.37 of 51) was higher than that of the original Australian, North American, Chinese, and Egyptian/Tunisian patient cohorts (Saleh et al., 2019, Sebaratnam et al., 2013, Sebaratnam et al., 2015, Yang et al., 2017). This difference is partly due to disease activity status. In our cohort, all patients were in relapse with active disease, but in the original English and other versions, most patients were in remission. Our patients' diagnosis was exclusively PV, but the original Australian cohort consisted of all varieties of AIBDs, with 40% of patients having PV (Sebaratnam et al., 2013). We also found worse QoL in women.

Our in-depth analysis suggests a good internal consistency, with Cronbach's alpha of .918. The test–retest reliability was good, with intraclass correlation coefficient of .885. Thus, the Persian version of ABQoL questionnaire is a valid and reliable instrument to monitor the quality of life of patients with PV. This tool can provide an estimate of the disease status and the effectiveness of different treatments. In previous studies, the disease-specific ABQoL was shown to be more sensitive than other instruments, such as the general dermatologic DLQI questionnaire (Patsatsi et al., 2017). General inquiries are made up of items that cover all situations with cutaneous or mucosal involvement and not necessarily the domains with the most importance in bullous conditions (Frew and Murrell, 2010). Krain et al. (2019) recently showed that the Skindex-S and ABQoL questionnaire are of the most use in clinical trials and patient management.

The internal consistency of the Persian version was higher than the original questionnaire in Australia and further validations in the Chinese and Egyptian/Tunisian cohorts (Saleh et al., 2019, Sebaratnam et al., 2013, Yang et al., 2017). Patient population may explain this superiority based on the data acquired. Other causes of such a discrepancy could be the cultural and educational status of the target populations. The test–retest reliability in our version of the ABQoL questionnaire was also close to that of other versions previously published (Kalinska-Bienias et al., 2017, Patsatsi et al., 2017, Saleh et al., 2019, Sebaratnam et al., 2013, Sebaratnam et al., 2015, Yang et al., 2017) and indicated consistency in results under similar conditions during further evaluations of quality of life.

Considering the construct validity of the ABQoL questionnaire, the original version showed three factors that explained 52.7% of the cumulative variances of all 17 items (Sebaratnam et al., 2013). In the Chinese version, three factors were also responsible for 53.5% of the variances (Yang et al., 2017). In both of these versions, the following three factors were identified: symptoms, and psychosocial, and mucosal. However, based on our factor analysis, we found two factors (mucosal-psychological and symptom-social) that explained 54.9% of the variances. In previous reports, there were disparities between the questions within each factor. For example, in the original English version, question 8 (effect of blistering disease on the enjoyable foods and drinks) was a mucosal factor, but in the Chinese version (Yang et al., 2017), this question was a component of the psychosocial factor. The cultural differences between the target populations explained this kind of disparity. Interestingly, we observed that patients with mainly mucosal complaints had psychosocial problems, and those with cutaneous symptoms had social difficulties.

In our factor analysis, one question (number 4) regarding slow skin healing did not fit any category, although it was fairly loaded on the second factor (social-symptoms). Defining a third factor for just one item was not methodologically meaningful, so we decided to put the question with the social-symptoms factor. In fact, by omitting this item, the Cronbach's alpha increased for the social-symptoms factor. Thus, one would think of deleting this item in the Persian version or at least changing the item to suit the Iranian population better. This issue could be due to many not paying attention to the healing pace or having different definitions of slow. This item is strongly correlated to cultural and educational issues.

The large sample size of patients with PV and active disease is one of the major aspects of our study. Meanwhile, there are some limitations to our study. First, our patients did not complete general health and DLQI questionnaires. Therefore, we could not ascertain the sensitivity of the Persian ABQoL questionnaire compared with other tools. Second, our data were acquired from a single institution. Although our institution is a referral, public, dermatology-specific care hospital, the undesirable selection bias could still affect the validity of this tool for Persian-speaking populations in general. Third, unfortunately, we did not have information on the educational status of the participants. Fourth, our patient cohort exclusively consisted of patients with PV. Therefore, we cannot extrapolate the validity and reliability of the Persian ABQoL questionnaire to autoimmune bullous conditions other than PV.

Before the development of disease-specific studies, general health and dermatologic questionnaires showed a significant deterioration in the quality of life of patients with AIBDs, especially PV. This decreased quality comprised both physical and psychological aspects of life, including anxiety and depression (Ghodsi et al., 2012). The downside of these instruments was their lack of coverage of specific domains in quality of life and inferior sensitivity toward changes during treatments contemplated in bullous diseases. The emergence of the Persian ABQoL questionnaire is a new useful tool to overcome these shortcomings. More studies are needed to assess the correlation of the ABQoL questionnaire to disease activity indices and other measurements of quality of life. Also, considering the diverse treatment-associated adverse effects, validation of the translated version of the treatment of autoimmune bullous disease quality of life questionnaire is suggested (Tjokrowidjaja et al., 2013). We would be able to measure the specific impacts of treatments on patients' quality of life.

## Conclusion

The Persian version of the ABQoL questionnaire is a valid and reliable tool to monitor quality of life in patients with PV. Further studies are needed to assess the sensitivity of this instrument to changes in different disease activity and the correlation with more general tools for the measurement of quality of life.

## Financial Disclosures

None.

## Funding

This study was partly supported by a grant from the National Institute for the Medical Development, Tehran, Iran (ref: 943673).

## Study Approval

N/A.

## Conflict of Interest

None.
